# Mental health law in Nepal

**DOI:** 10.1192/bji.2021.52

**Published:** 2022-02

**Authors:** Rakesh Singh, Seema Khadka

**Affiliations:** 1 Independent Mental Health Researcher, and Visiting Faculty Member, Department of Public Health, KIST Medical College, Tribhuvan University, Kathmandu, Nepal. Email: rakes4r@gmail.com; 2 Alumnus Public Health Student, School of Public Health, Patan Academy of Health Sciences, Lalitpur, Nepal

**Keywords:** Mental health, mental health law, mental health legislation, mental health policy, Nepal

## Abstract

During the past three decades Nepal has gone through series of reforms to address the mental health needs of the Nepalese population by promulgation of an exclusive National Mental Health Policy and related Strategic Action Plan. Small but significant improvements have been achieved in Nepal with regard to mental health policies and plans. This article discusses the evolution of mental health policies in Nepal and analyses the challenges to be overcome for their effective implementation.

## Background

Mental illness accounts for 7% of the global burden of disease as measured in disability-adjusted life years and 19% of all years lived with disability.^1^ Many low- and middle- income countries (LMICs) lack high-quality mental health services and have a higher prevalence of misdiagnosis and symptomatic treatment. The median number of psychiatrists per 100 000 populations is only 0.1 in LMICs.^2^ Although mental health policy is a vital tool, only 60% of member countries of the World Health Organization (WHO) have mental health policies; 71% have mental health plans; and 59% possess mental health legislation.^2^ The population covered by mental health legislation is very low in LMICs (36%) compared with high-income countries (92%).^2^

## Mental health in Nepal

The first epidemiological survey in Kathmandu, the capital city of Nepal, in 1984 showed that around 14% of the city's population had a mental illness. A pilot study of the National Mental Health Survey in 2018 reported the prevalence of mental disorders to be 12.9%^3^ and the actual burden is expected to be more at national level. However, there is only one public psychiatric hospital in Nepal. Moreover, there has been no significant rise in the mental healthcare budget for many years: it accounts for less than 1% of total national health expenditure, with the major proportion directed towards the mental hospital. To improve mental health and reduce the burden of mental disorders, a high-quality mental health service achieved through promulgation of mental health policies is required.

In 1975, the WHO stated that detection and management of mental disorders should be the tasks of primary healthcare workers. In 2015, the United Nations’ Sustainable Development Goals prioritised mental health and its promotion. In this regard, Nepal has shown commitment to attainment of a basic level of mental healthcare for Nepalese citizens. Nepal's 15th Five-Year Plan (2019–2024) includes provision of access to mental health service for everyone. The Epidemiology and Disease Control Division of the Department of Health Services was designated as a focal unit to oversee mental healthcare in 2018 and mental health programmes were operationalised by the Non-Communicable Disease and Mental Health Section. Despite challenges and barriers, significant incremental improvements have been achieved in Nepal with regard to mental health by endorsement of the policies and strategies in different periods.

## The 1996 National Mental Health Policy

In 1996 a comprehensive mental health policy was formulated and incorporated in the 9th Five-Year Plan. Its main goal was to provide at least a minimum level of mental healthcare to all Nepalese. The key components of the plan were: ensuring availability and accessibility of minimum mental health services for all; training human resources in mental healthcare; protecting the fundamental rights of mentally ill citizens; and improving awareness about mental health. The strategies adopted were decentralisation of services, integration of mental health with general health services and enhancing community participation. Integration of mental health services into basic health services to be delivered through primary healthcare was an important aspect of this policy, but limited data regarding service delivery in primary healthcare was a challenge to achieving this component.^4^ In addition, psychosocial aspects of healthcare were not addressed adequately. Despite various efforts, implementation of the policy remained ineffective and the Mental Health Act never came into existence.

Many years after the policy formulation, in 2017 the Ministry of Health and Population (MoHP) developed the Community Mental Health Care Package as a means of easing the policy's implementation. Likewise, other plans and strategies, such as the Multisectoral Action Plan for the Prevention and Control of Non-Communicable Diseases (2014–2020), the Nepal Health Sector Strategy (2015–2020) and the Nepal Health Sector Programme-II Implementation Plan (2010–2015), have included mental health, but were endorsed only very recently and remain partially implemented.

## The 2017 draft Mental Health Policy

In 2017 the MoHP drafted a mental health policy in line with Nepal's constitution, ensuring the right of every Nepalese citizen to mental and psychosocial health and guaranteeing the right to remain mentally sound and live a dignified life. The aims of the new draft were to ensure readily available and accessible basic mental health services for all; train the necessary workforce to deliver mental health and psychosocial services; protect the fundamental human rights of people with psychosocial disability and mental illness; promote public awareness of mental health and combat stigma against mental illness; and develop and manage health information systems and research. It proposed allocation of funding at federal and provincial level based on burden of mental illness, to be used for promotional, preventive, and remedial and rehabilitation activities. It envisaged a mental health division set up under the MoHP and the establishment of a separate mental health unit in each government-run hospital. Mental healthcare facilities would play a coordinating role, engaging with the community to promote mental health and ensure that people's mental health needs were assessed and addressed.^5^ Although the components of this draft policy were pertinent and were anticipated to be promising for improving the nation's mental health, it was not passed by the cabinet of ministers.

## The 2020 Mental Health Strategy and Action Plan

As mental health-related policy, plans and strategy were incorporated in the 2019 National Health Policy, the 1996 Mental Health Policy was automatically cancelled. There was therefore a need for a detailed strategy and action plan to address the challenges and problems in the mental health sector, so the 2020 National Mental Health Strategy and Action Plan was prepared. Its vision is to improve the mental and psychosocial health of Nepalese, enabling them to live productive and quality lives. The guiding principles of the plan are to ensure easy and equal access to high-quality mental health services; integrate mental health services into primary healthcare; maintain participation, cooperation and partnership between government, non-government and private sectors; and provide an evidence-based and comprehensive mental health service that is rights-based, participatory and inclusive. Its strategies include managing the necessary resources, workforce and delivery of mental and psychosocial services; conducting awareness campaigns to remove superstitions and myths related to mental illness and promote mental health; protecting human rights of people with mental illness and psychosocial disability; and promoting research by integration of mental health service-related information into the current information system. It also mentions monitoring and evaluation of programme implementation at all three government tiers – central, provincial and local.

The components of this plan seem to be propitious but the existence of only one psychiatric hospital in Nepal hinders accomplishing its goals. Although there are other referral hospitals providing psychiatric services, most are located in urban areas and lack adequate human resources.^6^ The idea of integrating mental healthcare into the primary healthcare system has already been promoted by the 1996 Mental Health Policy and Nepal Health Sector Programme-II. But the lack of mental health governance mechanisms at the national and district level has not allowed the policy provisions to be put into practice.^7^ Moreover, healthcare workers are already overstretched and this integration of services could further burden them. Despite these various barriers, integration can be achievable based on different enabling factors, such as constitutional provision for health as a human right, inclusion of mental health in the national five-year health plan, and inclusion of mental healthcare in the Multisectoral Action Plan for the Prevention and Control of Non-Communicable Diseases.

## Challenges and way forward

Factors including an insufficient workforce, limited training and frequent transfer of healthcare workers that limits those trained in mental health from providing continuing care are further barriers to attaining the goals of the Strategic Action Plan on mental healthcare. Furthermore, many people avoid seeking mental healthcare, largely because of stigma, discrimination and the high out-of-pocket costs of psychiatric care and medicines. The plan focuses on coordination with the health insurance programme to provide super-specialised mental health services at community level along with a telemental health service. However, this will be a challenge, as the national health insurance system is in its juvenile stage and there is inadequate digital health literacy among Nepalese people, especially those living in rural areas. Therefore, enhancing both mental and digital health literacy might be an appropriate strategy to improve mental healthcare utilisation. It is essential that every healthcare facility has a psychiatric unit. Moreover, a task-shifting approach involving the training of all primary healthcare workers in mental healthcare might be a viable solution towards making mental health services available at the community level.^8^ Additionally, it is crucial to clarify roles and responsibilities at each level of government to improve accountability and transparency in mental healthcare and create good governance to monitor both telemental health services and mental healthcare in Nepal.

## Data availability

Data availability is not applicable to this article as no new data were created or analysed in this study.

## References

[1] Rehm J, Shield KD. Global burden of disease and the impact of mental and addictive disorders. Curr Psychiatry Rep 2019; 21(2): 10. 30729322 10.1007/s11920-019-0997-0

[2] World Health Organization. Mental Health Atlas 2017. WHO, 2017 (https://www.who.int/publications/i/item/9789241514019 [cited 25 June 2021]).

[3] Jha AK, Ojha SP, Dahal S, Sharma P, Pant SB, Labh S, Prevalence of mental disorders in Nepal: findings from the pilot study. J Nepal Health Res Counc 2019; 17: 141–7. 31455924 10.33314/jnhrc.v0i0.1960

[4] Zhou W, Yu Y, Yang M, Chen L, Xiao S. Policy development and challenges of global mental health: a systematic review of published studies of national-level mental health policies. BMC Psychiatry 2018; 18(1): 138. 29776356 10.1186/s12888-018-1711-1 PMC5960139

[5] Himalayan News Service. Govt drafts new mental health policy. Himalayan Times, 10 Apr 2017 (https://thehimalayantimes.com/kathmandu/government-drafts-new-mental-health-policy).

[6] Mishra SR, Khanal P, Khanal V. Sustained neglect in mental health during Nepal's crises. Health Prospect 2018; 17: 4–7.

[7] Upadhaya N, Jordans MJD, Pokhrel R, Gurung D, Adhikari RP, Petersen I, Current situations and future directions for mental health system governance in Nepal: findings from a qualitative study. Int J Ment Health Syst 2017; 11: 37. 28603549 10.1186/s13033-017-0145-3 PMC5465682

[8] Purgato M, Uphoff E, Singh R, Pachya AT, Abdulmalik J, van Ginneken N. Promotion, prevention, and treatment interventions for mental health in low- and middle-income countries through a task-shifting approach. Epidemiol Psychiatr Sci 2020; 29: e150. 32744223 10.1017/S204579602000061X PMC7458538

